# A Discovered Ducal Seal Does Not Belong to the Incorporation Charter for the City of Krakow Solving the Mystery Using Genetic Methods

**DOI:** 10.1371/journal.pone.0161591

**Published:** 2016-08-25

**Authors:** Tomasz Lech

**Affiliations:** Department of Microbiology, Faculty of Commodity Science, Cracow University of Economics, Krakow, Poland; Seoul National University College of Medicine, REPUBLIC OF KOREA

## Abstract

The Incorporation Charter for the city of Krakow, the former capital of Poland, is one of the most valuable documents stored in the National Archives in Krakow. The document, which was written in 1257 on parchment, grants Krakow the Magdeburg rights and regulates its legal, statutory, economic and settlement-related aspects. The Charter was placed in the National Register of the Memory of the World UNESCO programme in 2014. A ducal seal, considered to be the lost seal detached from the Incorporation Charter, was found in the sphragistic collection after nearly 500 years. Unfortunately, it was uncertain whether the seal in question was indeed the missing part of the document. The aim of the study presented below was to solve this mystery. For this purpose, the parchment on which the Incorporation Charter was written was compared with the fragment of the parchment attached to the discovered seal. The study involved the analysis of selected mitochondrial DNA sequences and additional analysis at the level of nuclear DNA using microsatellite markers in the form of 11 STR (Short Tandem Repeat) loci, to identify the species and individual whose skin had been used to make the parchment. This analysis revealed that seal and parchment was from different individuals and thereby discovered that the seal was never a part of the Incorporation Charter. The study is further an example of informative DNA preservation in cultural heritage objects.

## Introduction

**Fig 1:**
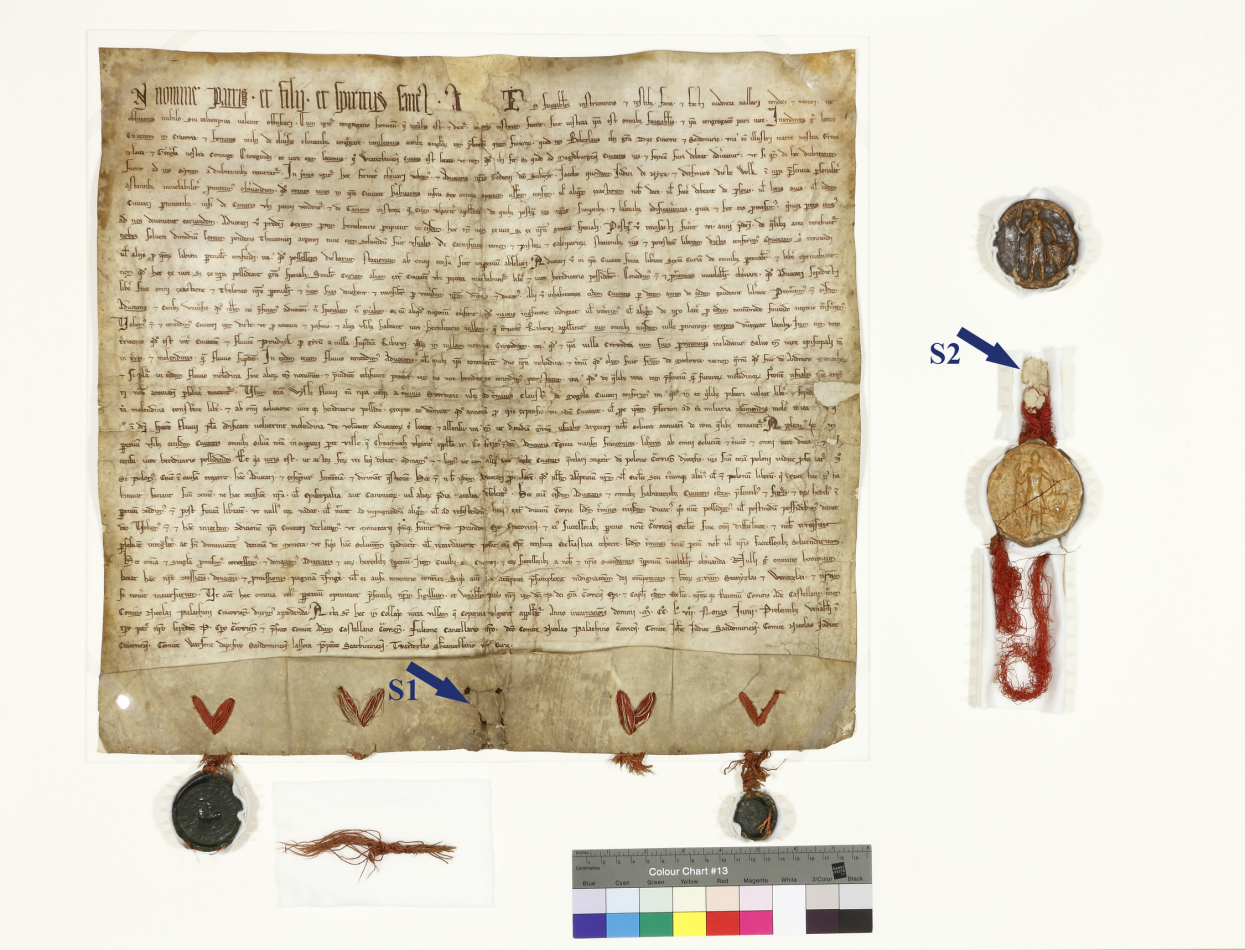
The Incorporation Charter for the city of Krakow. Arrows indicate the place of sampling, S1 –sample 1, S2 –sample 2.

The Incorporation Charter for the city of Krakow of 5 June 1257 is one of the most important and valuable documents of both Krakow and Poland. It is priceless and unique due to its content, form and technique with which it was made. It constitutes a foundation of Krakow’s history. The document clearly states the city’s origins and declares its organizational independence. It defines its territorial, social and cultural identity. The Charter is a monument of legal, diplomatic and literary culture and is a masterpiece of Medieval calligraphy and artistic craft. It is priceless for the Polish national heritage. The document is an inestimable resource for historical and technological research [1, 2]. The Incorporation Charter for the city of Krakow was written in Latin on a thick parchment, tanned on both sides. It is 505 mm high and 470 mm wide with a 74-mm-high pleat. This makes it one of the largest ducal parchment documents in Poland (Fig 1). It was affixed with 5 seals of Krakow castellan Adam, province governor Nicolaus, Duke Boleslaw the Chaste, Bishop Prandota and Krakow chapter. The fact that the incorporation privilege was authenticated by the most prominent people in the Duchy makes it even more unique. The only seals to have been preserved to the present day are the seals of the Krakow castellan and province governor. They were impressed in green wax and fastened to the document with ochre silk strings. The seals of the bishop and chapter have not been found. The only things that remained are fragments of ochre and cream-coloured strings. At that time, church seals were usually fastened to documents with double-coloured strings. Unfortunately, the most important seal, i.e. the one belonging to Duke Boleslaw the Chaste, has been detached from the document, leaving a hole in the parchment (Fig 1) [2]. Probably, the lost ducal seal was also attached to the document with silk strings.

The first account concerning the condition of the Incorporation Charter can be found in a transumpt of King Wladyslaw of Varna from 1440, which states that the document was intact. In the 16th century Behem Codex, the Incorporation Charter was entirely quoted, but its condition was not described. The presence of only four seals impressed in green wax and the lack of the fifth Duke’s seal was noted for the first time in the 17th century codex of Jan Zaleski. Subsequently, the Incorporation Charter for the city of Krakow was mentioned, among others, in a list of documents from 1802 prepared by Jozefant Wislincki. In another list, made by F. Piekosinski in 1874, there is a note stating that only two seals impressed in green wax were preserved, and the lack of the Duke’s seal was confirmed. In 1995–1997, during works concerning the sphragistic collection of the National Archives in Krakow, a seal of Boleslaw the Chaste was found–it was locked in a box number 74 (Fig 1). The broken round seal with a diameter of 64 mm is attached to a fragment of parchment with a red silk string. The seal presents the image of Duke Boleslaw the Chaste encircled by the following inscription: „+S`. BOLESLAI. DEI. GRA. DVCI[S]. [CR]ACOVIE. ET. SADOMIRIE.” [1]. Since the number of the box in which the seal was found and the number of the Incorporation Charter were the same (as stated in the document list from 1802), it was assumed that the seal was a part of the document. Moreover, this hypothesis was supported by the presence of a fragment of parchment attached to the seal and its defect in the Charter, which can be seen in Fig 1. That is why, the document is stored and occasionally exhibited with the said seal. The study presented below is an attempt to verify this hypothesis and provide the final answer to the question that has been intriguing conservators and historians.

Contemporary techniques and methods of molecular biology enable the investigation of historical objects and have been used to resolve various doubts. The literature broadly discusses issues associated with the possibilities offered by ancient DNA (aDNA) analysis. Ancient DNA can be extracted from historical objects, such as parchments or leathers, or from plant and animal remains (bones, teeth and hairs) [3–9]. Ancient DNA analysis can be used in technological studies involving objects of cultural heritage for: the identification of the animal which was used to make a given item, determination of their origin and authenticity, tracing cultural and technological development of past communities as well as learning about their social and religious rituals or even dietary habits [3, 4, 10, 11]. Species identification can be performed by analysing specific mitochondrial DNA markers, such as conserved cytochrome b fragment from all species, or by analysing selected sequences of mitochondrial control region obtained by designing species-specific primers [5, 6, 8, 11, 12]. However, the usage of more accurate and specific methods, such as the analysis of polymorphic short tandem repeats (STRs), enables examination of individual species by creating their own genetic profiles [9]. This method has been effectively used in various studies of ancient human and animal remains where DNA has been obtained from bones and teeth [13–16] or parchment [9]. It is also used in contemporary studies of biological fingerprints on paper [17], for tracing animal parentage [18], paternity testing and many more. Burger *et al*. [9] in their study from 2000, showed that STR profile analysis is an appropriate method for determining whether two parts of parchment used to be one and to provide information about a given animal and its population. The application of species-specific primers used in STR profiling has an additional advantage as it reduces the risk of contamination with other DNA [9].

The aim of the study was to verify whether the parchment of the Incorporation Charter for the City of Krakow and the fragment of the parchment attached to the discovered ducal seal had been made of the skin of the same animal. In other words, it was attempted to find out whether or not the two fragments of parchment used to be one.

For the purposes of species identification, the author analyzed conserved cytochrome b sequences amplified with the use of species-universal primers (expected product of 178 bp). This was verified by the analysis of sequences within the mitochondrial control region using primers specific for bovine DNA in position 16633–16810 (177 bp). Moreover, the analysis of sequences within the mitochondrial control region provided an answer to the question whether the parchment of the Incorporation Charter originates from an animal of the same species and haplotype as the parchment of the seal. However, considering the fact that mitochondrial DNA is more susceptible to contamination and that nuclear DNA analysis is characterized by higher discrimination power [9, 19], it was decided to supplement the study with an additional analysis of genetic markers within nuclear DNA. That is why the investigation involved the analysis of polymorphism of 11 selected STR loci, which included sequences located on 10 chromosomes (1, 7, 10, 12, 14, 16, 18, 19, 21, 26) of the bovine genome. The STR loci were selected on the basis of literature reports, and the length of amplified sequences ranged from 74–125 bp.

## Results

**Fig 2:**
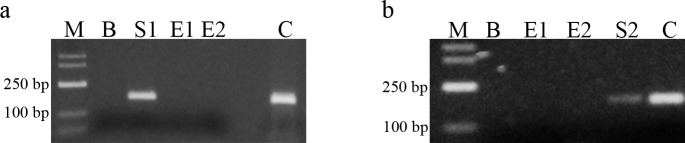
Electrophoresis of PCR products for cytochrome b. a–Sample 1 extraction; b–Sample 2 extraction; M–DNA size marker; B–blind PCR sample; E1, E2, E3, E4 –blind samples during extraction; C–positive control; S1 –Sample 1; S2 –Sample 2.

The spectrophotometric analysis of DNA concentration revealed the values of 4.4 ng/μl for Sample 1 and 3.1 ng/μl for Sample 2. Following DNA amplification and purification, its concentration amounted to 78.5 ng/μl and 56.3 ng/μl, respectively. Moreover, the quality of the genetic material was confirmed by obtaining PCR products for cytochrome b, which also confirmed the lack of contamination of blind samples (Fig 2).

PCR product sequencing for cytochrome b with universal primers resulted in the identification of the following species in the NCBI database: Bos taurus (99% similarity of the obtained sequence to the sequence in the GenBank, accession number KM507199.1) for Sample 1 and Bos taurus (86%, GenBank accession number JX472274.1) for Sample 2 (S1 File). Based on this identification, it was decided to analyse the samples further in terms of bovine DNA. The next stage of the study invovled species identification with the use of primers (178F and 309R) specific for bovine mtDNA control region [5]. Sequencing revealed that Sample 1 had been made of bovine skin of the Bos taurus with haplotype 14 (99%, GenBank accession number KR857532.1) and Sample 2 was also bovine of the Bos taurus with haplotype 16 (98%, GenBank accession number KR857534.1) (S1 File). The analyses of universal sequences for cytochrome b and species-specific sequences for mitochondrial control region revealed that both parchment samples had been made of bovine material. The results obtained in the mitochondrial control region analysis also demonstrated that the two fragments of parchment were made of animals with different mitochondrial halotypes, which provided evidence that they never originated from the same parchment. Nevertheless, the results were verified with nuclear DNA analysis due the significance and unique nature of the tested item.

In order to find out whether or not the two parchment samples originated from the same animal, the STR sequence polymorphism was analysed. The products of each marker were amplified in separate reactions so as to adjust reaction conditions as precisely as possible to each pair of primers. The optimization of PCR conditions is significant for the amplification of difficult and short sequences, particularly when working with severely degraded genetic material. Moreover, the application of appropriate PCR conditions helps obtain specific products and minimises the risk of obtaining non-specific products. Table 1 presents the results of the analysis of selected microsatellite sequences. Of 11 STR loci tested, appropriate PCR products for both samples were obtained in 9 cases. Different results (in terms of the length of PCR products in bp–the number of tandem repeats) were obtained for seven markers, i.e. TGLA227, TGLA57, TGLA176, BM2934, BM6122, CSRM60 nad INRA35, which differentiated S1 from S2. This proved that the two fragments of parchment had been made of two different individuals (Table 1). The value of markers UWCA4 and RM026 was found to be the same for both samples. Poor quality of the PCR products obtained for markers INRA117 and ETH3, however, did not allow for their analysis after capillary electrophoresis and therefore they were rejected. The individual DNA profiles for both samples indicate that the parchments in question were made of the skin of two different individuals and did not constitute one parchment. It must be noted that forensic analyses using DNA profiles including 7 STR loci enable one to confirm or exclude the common origin of two genetic samples.

**Table 1:** Results of STR sequence analysis.

Microsatellite	Sample 1 STR products [bp]	Sample 2 STR products [bp]
TGLA227	92/94	86
TGLA57	92	80/84
UWCA4	75	75
INRA117	92/94/97/99	-
TGLA176	79	81
BM2934	86/107	84/86
RM026	92/94	92/94
ETH3	-	-
BM6122	91/93	82/84
CSRM60	79/87	79/81
INRA35	109/111	90

## Discussion

The analysis of ancient DNA is a highly promising method for solving various archaeological, social and population mysteries. Unfortunately, it has certain limitations, mainly associated with a degree of DNA degradation in a given sample. This is affected by the type of material (parchment, skin, bones), its preservation state, storage conditions or the presence of PCR inhibitors that can prevent further analyses. Due to the level of ancient DNA degradation and its short fragments, it is essential to ensure appropriate conditions while working with such materials in order to minimise the risk of DNA contamination with contemporary or different ancient DNA [4, 5, 9, 14, 20]. Despite numerous factors that can contribute to the degradation of parchment aDNA, it is still possible to extract it and use it for further analyses. As stated in the literature, aDNA is usually severely degraded (100–200 bp) [5]. That is why scientists frequently choose mtDNA sequences or shorter nuclear DNA sequences for analyses [5, 10, 19, 21].

This study began with the identification of the animal whose skin had been used to produce the parchment. Studies of this type usually involve a traditional analysis with the use of light microscopy [22]. This, however, can be prevented by degradation of parchment, tanning, covering with dyes or insufficient size of the tested object [5]. In the case of the parchment which was used to write the Incorporation Charter for the city of Krakow and the fragment of the parchment attached to the seal of Duke Boleslaw the Chaste, the previous attempts to identify the species with this method were ineffective (unpublished data). That is why the analysis of selected mtDNA sequences was employed. This type of DNA is more easily retrieved from ancient materials because of the multiplicity of its copies and its resistance to degrading factors [10, 19]. The sequencing of products obtained with the use of universal cytochrome b primers and primers specific for bovine mitochondrial control region revealed that the parchment of the Charter had been made of bovine material. Similarly, the fragment of parchment at the seal was also made from bovine skin. The selection of the sequences for species analysis occurred to be accurate, and it was dictated by studies in which these markers were successfully used for aDNA analysis [4–6, 23, 24].

As has already been mentioned above, the main aim of this study was to verify the hypothesis that the seal of Boleslaw the Chaste, found in the 1990s, used to be a part of the Incorporation Charter for the city of Krakow. In other words, it was attempted to find out whether the fragment of parchment at the seal and the parchment of the document had been made of the skin of the same animal. The analysis of miotochondrial control region sequences demonstrated that the parchment samples were made of animals with different mitochondrial haplotypes. This was the basis for a conclusion that the parchment samples were made of two different individuals of the same species.

Although the efficacy of mtDNA investigation in aDNA analysis is higher, nuclear DNA analysis is characterised by superior sensitivity and discrimination power [19]. The analysis of STR profiles in nuDNA is broadly used for various purposes, e.g. in forensic studies when searching for biological traces, animal population studies or examining ancient DNA [9, 14, 25, 26]. Thanks to advanced technology, the analysis of STR sequence polymorphism has become a powerful tool for personal identification. The probability that two individuals with the same profile of 6 STR loci exist is 1 to 50 million, which guarantees high sensitivity of this method. Moreover, the usage of this method for genetic profiling of animals is becoming more and more popular [25, 26]. Burger *et al*. [9] presented an experiment in which they showed that the analysis of 7 selected STR sequences specific for bovine DNA can prove that two parchment fragments used to be one and had been made of the skin of the same animal. That is why it was also decided to conduct a similar analysis in this study. Based on the analysis of 9 of 11 STR sequences on 10 chromosomes, it was possible to conclude that the two samples tested had been made of two different individuals. To sum up, aDNA analyses using both mtDNA and nuDNA markers demonstrated that the two fragments of parchment were made of bovine skin of two different individuals. This means that the parchment of the Incorporation Charter and the fragment of the parchment attached to the seal were never parts of the same parchment, and that the discovered seal of Boleslaw the Chaste was never an integral part of the Incorporation Charter for the city of Krakow.

The success of ancient DNA analysis depends on its preservation in biological materials. This, in turn, is associated with multiple factors, which have been partially discussed above. However, proper storage conditions, e.g. low temperature, low humidity, no UV radiation, no degrading microorganisms or rapid hide tanning after slaughtering an animal, seem to play the crucial role. Studies have shown that storage conditions, and not the storage duration, is responsible for aDNA quality [27]. As historians report, the document in question was stored in a locked box in cabinets or treasuries of archives, and only its copies were used for administrative purposes. It has never been exhibited in museums except for one-day occasional exhibitions [1], which has definitely had a positive influence on aDNA preservation.

## Conclusions

Animal skin was one of the first materials used by humans. It was initially used to produce clothes and tools, and later, it served as parchment thanks to which it became the carrier of culture [4]. From today’s perspective, parchments are an exceptionally interesting source of information for historians and conservators. It turns out that historical knowledge can be derived not only from information written or drawn on parchments. The genetic material hidden in parchment can also constitute a rich source of information. Thanks to the developments of science, we have at our disposal better and better tools for aDNA analysis that can enable better understanding of the past culture, habits of people and their migrations. Moreover, it can also help gain detailed information about items of cultural heritage, as it has been presented in the study above. It was a great success to first extract aDNA from historical parchments, particularly from the Incorporation Charter which is nearly 760 years old, and then to solve one of the most interesting historical mysteries associated with this document by analyzing genetic markers.

## Materials and Methods

The test samples were obtained from historical parchments. Both historical objects are stored in the headquarters of the National Archives in Krakow under specimen numbers “*Perg2”* for the Incorporation Charter and “*Luzne pieczecie 111*” for the seal. In order to protect the Incorporation Charter, this document is available to other researchers and scientists. The micro- and macroscopic analyses have shown that the document was made of a single fragment of parchment. As has been mentioned above, only two seals with silk strings are currently attached to the parchment. Double-coloured strings are the only remnants of other two seals. The main ducal seal was detached from the document together with its string and a fragment of parchment. A hole in the parchment can still be seen (Fig 1). The discovered seal, together with a string and a fragment of parchment is also presented in Fig 1. The first sample was obtained from the fragment of the parchment attached to the seal (Sample 1), and the second sample was collected from the Incorporation Charter for the city of Krakow (Sample 2) (Fig 1). In order to avoid contamination, the two samples were collected using sterilized equipment with an interval of 4 weeks. Moreover, further analyses were conducted in two different places, i.e. in the Genetic Laboratory of the Department of Microbiology at the Cracow University of Economics and in the Molecular Biology Laboratory of the Department of Genetics and Evolution at the Jagiellonian University in Krakow, Poland.

### DNA extraction and PCR amplification

In order to minimise the risk of sample contamination, necessary safety and hygiene measures were undertaken. All work associated with the genetic material was conducted in gloves, masks, caps and protective aprons. Prior to extraction, workbenches and lab rooms were adequately prepared, cleaned and disinfected (washed with ethanol and UV-radiated). In the laboratories where DNA extraction was conducted, bovine genetic material had never been tested before. Prior to genetic material extraction, both parchment samples were gently cleaned using 70% ethyl alcohol. Ancient DNA was extracted with the use of a commercial DNeasy Blood & Tissue kit by Qiagne (Hilden, Germany). The samples were incubated in accordance with the protocol in 180 μl of buffer ATL with 20 μl of proteinase K for approximately 4h at a temperature of 56°C in a thermoshaker. After complete sample dissolution, 4 μl of RNase A (100 mg/ml) was added and the sample was incubated for 2 minutes at room temperature. Subsequently, following the addition of 200 μl of buffer AL and 200 μl of ethanol (96–100%), and mixing thoroughly by vortexing, the mix was transferred into a DNeasy mini spin column. Following column rinsing, DNA elution was performed: 2x with 50 μl of buffer AE. Moreover, during the extraction from each sample, the same procedure was conducted for two samples without genetic material. The concentration of the obtained DNA was measured with the use of the NanoDrop 2000c UV-Vis Spectrophotometer (Thermo Fisher Scientific, Wilmington, USA).

Extracted DNA from the Charter parchment and from the seal parchment was amplified with the use of the GenomePlex® Whole Genome Amplification kit for its multiplification. Samples for negative control during the DNA extraction procedure were also amplified with the GenomePlex® Whole Genome Amplification kit. Subsequently, the fragmentation buffer was added to the DNA suspension with a concentration of 1 ng/1μl and it was incubated at 95°C for exactly 4 minutes. Next, a library of fragments was created by adding the library preparation buffer, library stabilization solution and, following incubation as required in the protocol, library preparation enzyme. Subsequently, it was incubated in a thermal cycler as recommended by the manufacturer. By adding the amplification master mix, WGA DNA polymerase and water to the mixture, a master mix for final DNA amplification was obtained. DNA amplification was conducted according to the programme of initial denaturation at 95°C for 3 minutes and 14 cycles of as follows: denaturation at 94°C for 15 seconds and annealing/extension at 65°C for 5 minutes. The manufacturer recommends the kit as a sensitive and efficient tool for genetic material amplification which ensures accuracy of reactions and prevents cross contamination. The kit is also recommended for difficult and degraded DNA extracted from formalin-fixed and paraffin-embedded tissue. The components of the WGA kit do not contain Bovine Serum Albumin, or at least no such information is provided by the manufacturer.

The polymerase chain reaction with universal primers for cytochrome b was conducted in 25 μl of the reaction mix consisting of 3.5 μl of DNA template, 0.4 μmol of each primer (Table 2) (Genomed, Warsaw, Poland); 200 μl/l of each dNTP (Sigma-Aldrich, USA), 2 U of Taq DNA polymerase (Invitrogen, Thermo Fisher Scientific), 1x polymerase buffer and 1.5 mmol of MgCl_2_. The reaction was conducted in a T100 thermal cycler (Biorad, Hercules, CA, USA) according to the programme that consisted of: initial denaturation at 95°C for 5 minutes, 40 cycles with a denaturation at 95°C for 30 s, annealing for 30 s at a temperature of 55°C, elongation at 72°C for 1 minute, and final elongation at 72°C for 10 minutes. The amplification with primers specific for bovine mitochondrial DNA control region (Table 2) was conducted in an identical reaction mix by following a similar amplification programme with annealing temperature of 66°C.

**Table 2:** PCR primers used in this study.

Gene target	Primer sequence	Amplicon size [bp]	Reference
Universal primers for cytochrome b	Uni-Fw–TCCCCAACAAACTAGGAGG; Uni-Rv–ACTGGTTGTCCTCCAATTCA	178	[6]
Bos taurus—mtDNA—mitochondrial control region in position 16633–16810	178F –GCCCCATGCATATAAGCAAG; 309R –GCCTAGCGGGTTGCTGGTTTCACGC	177	[5, 28]

The PCR products obtained were separated by electrophoresis in agarose gel with a concentration of 1.8% and visualized under UV light using a Simply Safe dye (Eurx, Poland).

### DNA sequencing

Amplicons for cytochrome b and for primers specific for bovine mitochondrial control region (178F and 309R) were purified and sequenced (Genomed, Warsaw, Poland). Based on the obtained nucleotide sequence, species identification was conducted using the NCBI (National Centre for Biotechnology Information) database and the Basic Local Alignment Search Tool (BLAST) for DNA sequence analysis [29].

### STR sequence analysis

**Fig 3:**
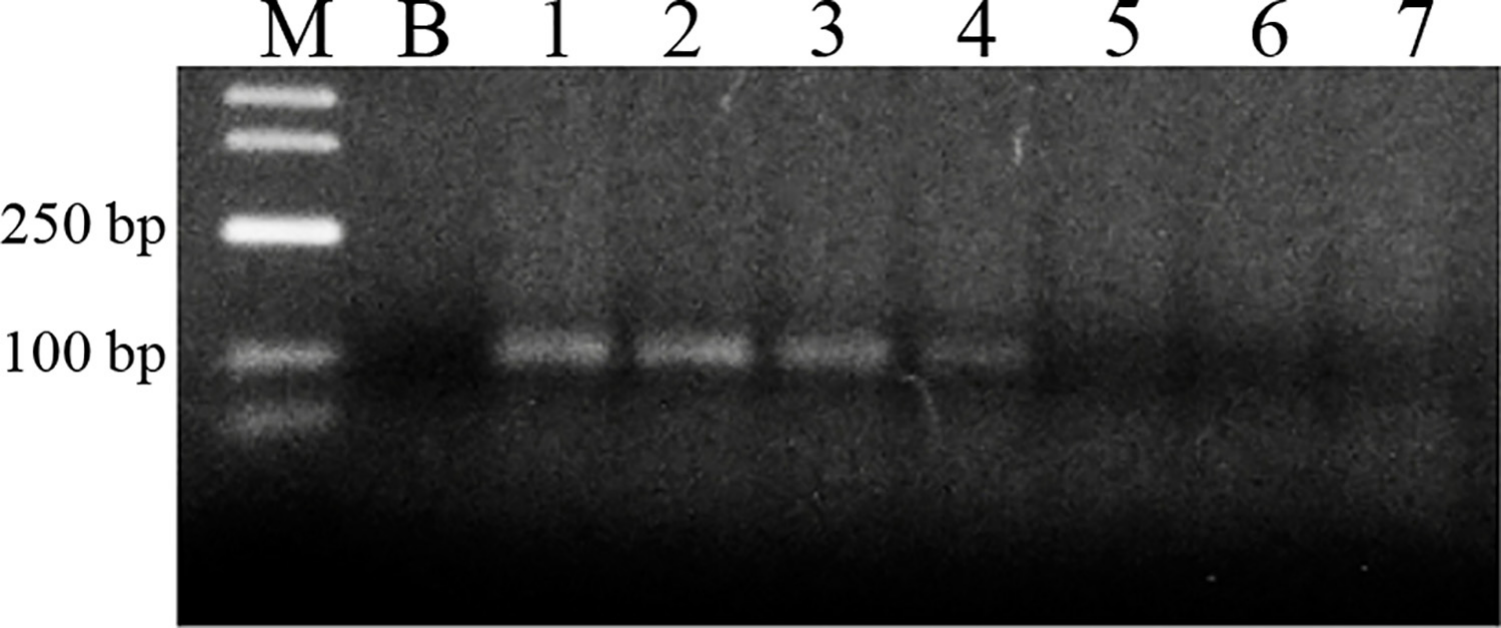
Example electrophoresis during experimental determination of STR primer annealing temperature. The image presents the product of TGLA227 marker, M–DNA size marker, B–blind sample, 1–7 –products amplified in thermal gradient of 51–58°C.

Amplifications of 11 STR loci (BM2934, BM6122, CSRM60, ETH3, INRA117, INRA35, RM026, TGLA176, TGLA227, TGLA57 and UWCA4) were conducted separately for each sample (Table 3). These loci were selected on the basis of the literature reports [9, 30–35, 36] and FAO recommendations concerning the characterization of animal genetic resources [37]. The amplification was conducted in 25 μl of the reaction mix consisting of 3.5 μl of DNA template, 0.5 μmol of each primer (Table 3) (Genomed, Warsaw, Poland), FAM-labelled forward primers, 200 μl/l of each dNTP (Sigma-Aldrich, USA), 2 U of Taq DNA polymerase (Invitrogen, Thermo Fisher Scientific), 1 x polymerase buffer and 1.5 mmol of MgCl_*2*_. The reaction was conducted according to the program that consisted of: initial denaturation at 95°C for 5 minutes, 40 cycles with denaturation at 95°C for 30 s, annealing for 30 s at a temperature specific for each primer (Table 4), elongation at 72°C for 1 minute, and final elongation at 72°C for 10 minutes. The annealing temperature for each pair of primers was determined in tests using T100 thermal cycler gradient (Fig 3).

**Table 3:** STR information.

Marker	Chromo-some	Primer sequence	Amplicon length [bp]	Allele number	Refe-rence
BM2934	14	F: CCAATTGTCTTCCTAGCTCTTC; R: CTGTTAGTTCTGCCAAAATCCC	81–103	9	[29]
BM6122	12	F: TTCTCTAGGCTTATCAGTGGCC; R: GGAGTTGCAAAGAGCTGGAC	77–103	7	[30]
CSRM60	10	F: AAGATGTGATCCAAGAGAGAGGCA; R: AGGACCAGATCGTGAAAGGCATAG	79–117	-	[36]
ETH3	19	F: GAACCTGCCTCTCCTGCATTGG; R: ACTCTGCCTGTGGCCAAGTAGG	105–125	9	[31]
INRA117	1	F: GTTTCTAGTAACATATTGAC; R: TTAGACATGACTGAAGCAAC	95–103	11	[32]
INRA35	16	F:ATCCTTTGCAGCCTCCACATTG; R:TTGTGCTTTATGACACTATCCG	100–124	7	[32]
RM026	26	F: TTGTACATTTCTGTCAATGCCTT; R: ACAATGTCATTGGTCAATTCATT	85–95	4	[30]
TGLA176	7	F: AGTAGGAATACCCCAGGAGGAA; R: TCAAGGCACAAGCACACAGTCA	74–92	6	[30]
TGLA227	18	F: CGAATTCCAAATCTGTTAATTTGCT; R: ACAGACAGAAACTCAATGAAAGCA	76–104	14	[33]
TGLA57	1	F: GCTTTTTAATCCTCAGCTTGCTG; R: GCTTCCAAAACTTTACAATATGTAT	86–102	8	[33]
UWCA4	21	F: CAGCTTGAAATGAGTATGTCACC; R: CAACAAGAAAGCAGAGACTCC	75–93	6	[30]

**Table 4:** STR annealing temperature.

Marker	Annealing temperature [*°*C]
BM2934	58
BM6122	58
CSRM60	60
ETH3	59
INRA117	54
INRA35	60
RM026	58
TGLA176	58
TGLA227	52
TGLA57	53
UWCA4	52

One microliter of each obtained PCR product was used to conduct capillary electrophoresis with a ROX size standard. The results were analyzed with the use of the Gene Mapper v 3.2 (Applied Biosystems)

## Supporting Information

S1 File (DOCX)
