# Supplementary material for: A Discovered Ducal Seal Does Not Belong to the Incorporation Charter for the City of Krakow Solving the Mystery Using Genetic Methods
Source: PLoS One. 2016 Aug 25;11(8):e0161591. doi: 10.1371/journal.pone.0161591 (PMC4999217; doi:10.1371/journal.pone.0161591)
Supplement: S1 File — (DOCX) [file pone.0161591.s001.docx]

Suplementary

Sequences obtaind for cytochrome b with primers Uni-Fw and Uni-Rv:

Sample 1

5'-TCCCCAACAAACTAGGAGGGSARWKGKACTCTCWTCTAATTCTTGCTCTATCCCCCTACTACACACCTCCAAACAACGAAGCATAATATTCCGACCACTCAGCCAATGCCTATTCTGAGCCCTAGTAGCAGACCTACTGACACTCACATGAATTGGAGGACAACCAGTACTGGTTGTCCTCCAATTCA-3'

Sample 2

5'-TCCCCAACAAACTAGGAGGCAAGCATCTCTCATCCTATTCTWGCWCTATCCCCCTMCTMCAYAYMTCCAACAACGAAGCATAATATTCCGACCACTCAGCCAATGCTTTTCTGAKCCCTAGTAGCAGACCTCCTGWTCTCCMTGAATTGGAGGACAACCAGTACTGGTTGTCCTCCAATTCA-3'

Sequences obtaind for mitochondrial control region with primers 178F and 309R:

Sample 1

5'-GCCCCATGCATATAAGCAAGCSTAAYTAGCAGTAMTAATACATATAATTATTGACTGTACATAGTACATTATGTCAAACTCATTCTTGATAGTATATCTATTATATATTCCTTACCATTAGATCACGAGCTTAATTACCATGCCGCGTGAAACCAGCAACCCGCTAGGCGCCTAGCGGGTTGCTGGTTTCACGC-3'

Sample 2

5'-GCCCCATGCATATAAGCAAGCASTCCYTWGCAGTMATAATACATATAATTATTGACTGTACATAGTACATTATGTCAAATTCATTCTTGATAATATATCTATTATATATTTCTTACCATTAGATCACGAGCTTAATTACCATGCCGCGTGAAACCAGCAACCCGCTAGGCGCCTAGCGGGTTGCTGGTTTCACGC-3'
